# Investigation on Combustion Characteristics and Molecular Structures of Heiyanquan Mining Area, Xinjiang, China

**DOI:** 10.3390/molecules29061231

**Published:** 2024-03-10

**Authors:** Tong Feng, Qiang Zeng

**Affiliations:** 1 Center for Underground Coal Fire, College of Ecology and Environment, Xinjiang University, Urumqi 830046, China; fengtong1346@163.com; 2 Key Laboratory of Oasis Ecology of Ministry of Education, Xinjiang University, Urumqi 830046, China

**Keywords:** coal spontaneous combustion, molecular structure, functional group, coal molecular model

## Abstract

In order to comprehend the molecular composition of coal and better understand the process of coal combustion, this study involved the development of a molecular structure model for Heiyanquan coal in Xinjiang, as well as the optimization and annealing dynamics simulation of the model. Thermogravimetric analysis (TG), Fourier transform infrared spectroscopy (FTIR), and high-resolution transmission electron microscopy (HRTEM) were utilized to investigate the spontaneous combustion characteristics of coal at different temperatures (room temperature, 50–500 °C with 50 °C interval). The findings revealed that the coal primarily consists of aromatic carbon, with the aromatic structure mainly comprising naphthalene, anthracene, and phenanthrene, and the aliphatic carbon mainly consisting of CH_2_ and CH, along with a small quantity of minerals. The empirical molecular formula of Heiyanquan coal was determined to be C_175_H_125_O_21_N_3_. After the optimization, the total energy of the model was significantly reduced, and the aromatic layers tended to align in a regular parallel manner, with van der Waals energy playing a crucial role in maintaining structural stability. As the temperature increased, the activation energy of the three stages also increased, with the combustion stage exhibiting the highest activation energy. The presence of hydroxyl groups and oxygen-containing functional groups was found to mainly participate in the reaction, while the content of aromatic hydrocarbons remained relatively stable, C=C exhibited a decreasing trend, and C-O displayed an increasing trend. Moreover, it was observed that 1 × 1 and 2 × 2 were the predominant aromatic stripes in the coal samples, accounting for more than 90% of the total stripes.

## 1. Introduction

Fossil fuels such as coal are the primary source of energy [1,2], and coal fires are a hazard associated with coal mining, while spontaneous combustion of coal can result in resource loss and environmental pollution [3,4]. Therefore, from a scientific perspective, the investigation of coal structure, the evolution of its characteristic parameters, and its microstructure during the oxidative combustion process holds great significance in examining the mechanisms of coal spontaneous combustion and preventing coal spontaneous combustion.

For the study of the molecular structure of coal, changes in the parameters of the spontaneous combustion characteristics and microstructure of coal, as well as studies related to the molecular dynamics of coal, several studies have been carried out from various coal seams with different ranks in several countries. Since the 1960s, various studies have proposed different models for the macromolecular structure of coal, such as Fuchs [5], Given [6], Wiser [7], Shinn [8], and other models. With the emergence of various modern analytical techniques and the improvement of molecular model construction methods, some scholars have used a combination of physical methods [9,10,11,12,13] and chemical methods [14,15] to establish molecular models of coal of different regions, to gain a deeper understanding of the reactions and structural properties of coals. Chai et al. [16] constructed a molecular model of Zhundong Wucaiwan coal by extracting information characterizing coal microstructural parameters using computer software and experimental data, and heating it to obtain the changes in its macroscopic and microscopic characteristics. Meng et al. [17] constructed a molecular model of coal by experimentally testing and analyzing the chemical structure of coal samples from Tunliu Mine, and used molecular simulation technology to achieve three-dimensional visual characterization and quantitative analysis of coal molecular pore structure. Ma et al. [18] constructed and optimized a macromolecular structure model of Fengxian coal in Shaanxi using ^13^C-NMR, FTIR, and XPS, which provided a model basis for the study of the microcrystalline structure evolution of graphitization of high-grade coals on a molecular scale. Zhang et al. [19] constructed and optimized the molecular structure model of coal from the Qingdong coal mine and proposed another aggregate structure model. Zhang et al. [20] constructed a molecular structure model of coal from the Zhalute region in Inner Mongolia, and used molecular dynamics simulation to study the changes of anthracite coal in the Zhalute region from the molecular scale. Wang et al. [21] constructed the macromolecular structure of coal from the Zhundong region of Xinjiang and used FTIR, XPS, and TG to analyze the changes in the molecular structure of the coal under different inhibitor concentrations and temperatures, and to derive the optimal inhibitor concentration. The above studies have constructed the coal macromolecular structure in different regions using experimental and simulation methods, which helps to understand the molecular structural characteristics and chemical composition of coal, and from the molecular point of view, can improve the efficient use of coal, which is a breakthrough point for the development of the coal chemical industry.

Zhang et al. [22] investigated the macromolecular structure, microcrystalline structure, and oxidation process of coal from the Baishihu coal mine using XPS, ^13^C-NMR, and FTIR. Liang et al. [23] conducted qualitative and semi-quantitative studies on the functional groups of two different coal rank coals, long-flame coal and anthracite, based on FTIR spectroscopy and sub-peak fitting techniques, combined with sub-peak area fitting, and comparatively analyzed the chemical structural parameters of the two coals. Lei et al. [24] quantitatively characterized the streak length, orientation, and stacking distribution of Yangquan No. 3 anthracite using HTREM. Deng et al. [25] and Shi et al. [26] studied the reaction mechanism of spontaneous coal combustion. Gu et al. [27] compared the improvement of flame retardancy of TPU by biobased intumescent flame retardant (PAMAD) using FTIR/^13^C-NMR and other experiments. Kok et al. [28] investigated the non-isothermal thermogravimetric analyses and the kinetics of oil shale samples under different conditions.

While recent research has primarily focused on molecular modeling or combustion characterization of coal, there have been limited in-depth and comprehensive studies in this direction. This study selects coal samples from Heiyanquan as the research subject. The structural parameters of coal were obtained through proximate analysis, ultimate analysis, XPS, and ^13^C-NMR. Subsequently, geometric optimization and annealing kinetics simulation were conducted using the molecular simulation Materials Studio (MS) 2019 software. The coal was exposed to varying temperatures, and changes in activation energies, functional groups, and lattice fringe lengths of the aromatic layer at different combustion stages were measured by FTIR, TG, and HRTEM. This led to the construction of a molecular structure model of coal, aiding in comprehending the structural characteristics of Heiyanquan coal at the molecular level, understanding the combustion process’s reaction law, and providing assistance in preventing and treating coal sample spontaneous combustion.

## 2. Results and Discussion

### 2.1. Proximate and Ultimate Analyses

According to the proximate and ultimate analysis results, the atomic ratio of each element in the Heiyanquan coal sample was calculated, as shown in Table 1. The results showed low moisture and ash content, higher volatiles, highest fixed carbon content, with oxygen-containing functional groups accounting for a relatively large proportion and nitrogen-containing functional groups accounting for a very small proportion. The sulfur content was too low to be considered in the construction process of the coal molecular model.

### 2.2. XPS Test Results

**Figure 1:**
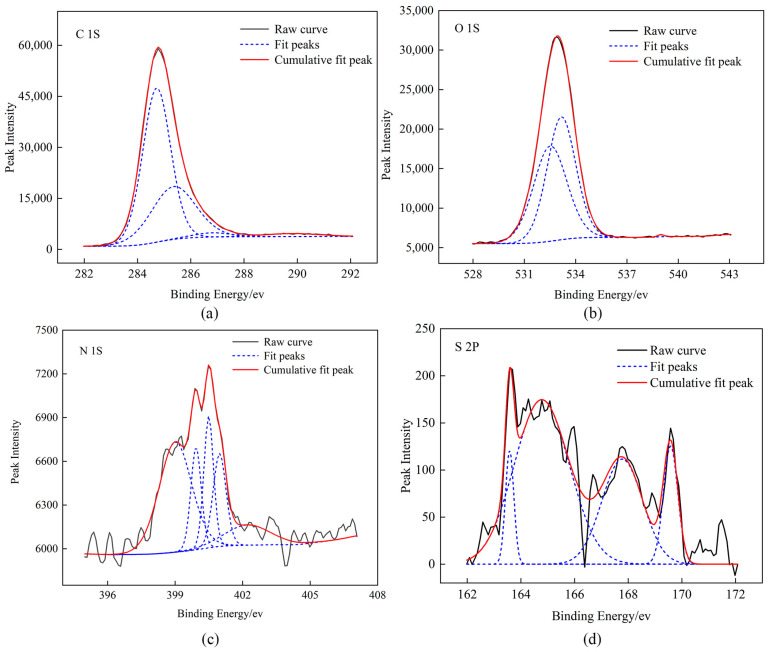
XPS peak-fitting spectra of raw coal, (**a**) C element (**b**) O element (**c**) N element (**d**) S element.

The elements C, H, O, N, and S of the coal samples existed in the form of various functional groups, and their XPS spectra were formed through the superposition of multiple peaks. Therefore, Advantage software was used to perform the peak-fitting analysis on their spectra [29]. The fitting diagram is shown in Figure 1. According to the affiliation of each binding energy, the functional group composition of each element was determined, and the results are shown in Table 2.

The existing forms of C (1s) in coal are C–C, C–H (284.72 eV), C–O, C–O–C (285.36 eV), C=O (286.81 eV), and COOH (289.83 eV) [30]. The calculation of the relative area of the sub-peaks showed that C in the coal samples was mainly aromatic carbon and its substituted alkanes, followed by ether bonds. The existing forms of O (1s) in coal are C=O (532.52 eV), C–O (533.17 eV), and adsorbed oxygen (538.98 eV). The existing forms of N (1s) in coal are pyridine nitrogen (N–6) (399.01 eV, 399.93 eV), quaternary nitrogen (N–Q) (402.23 eV), pyrrole nitrogen (N–5) (400.49 eV, 400.97 eV). The calculation of the relative area of the sub-peaks showed that the ratio of N–5 to N–6 was approximately 2:1and that the nitrogen element was mainly pyridine nitrogen. The existing forms of S in the coal are mercaptan thiophenol (163.58 eV), thiophene sulfur (164.77 eV), sulfoxide sulfur (167.78 eV), and inorganic sulfur (169.58 eV). The calculation of the relative area of the sub-peaks showed that the occurrence form of the sulfur element was mainly thiophene sulfur.

### 2.3. XRD Test Results

**Figure 2:**
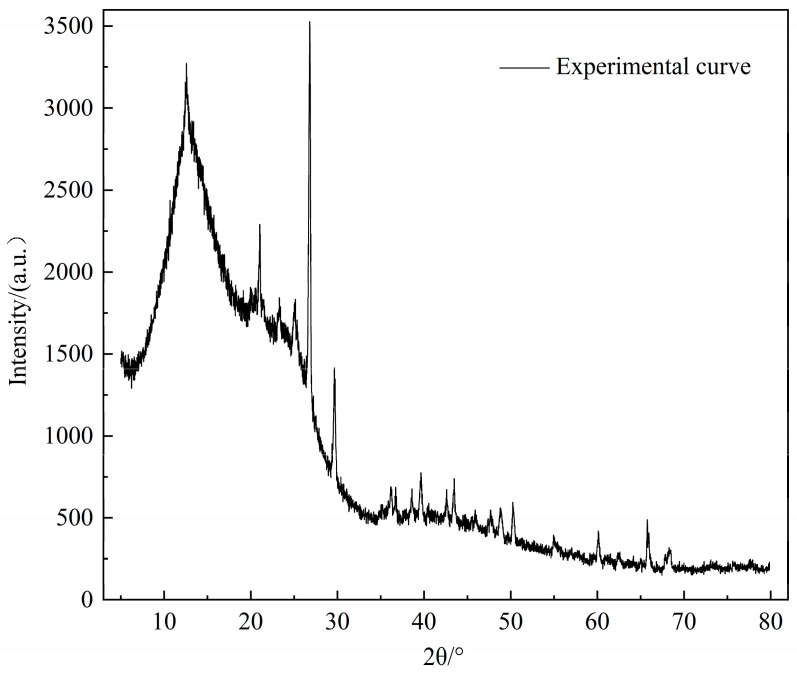
XRD spectrum of Heiyanquan coal.

XRD can test the crystal structure of substances and is also widely used to determine coal structure. The XRD spectrum of the Heiyanquan raw coal is shown in Figure 2. The analysis and calculation of the XRD spectra data can provide information on the aromatic lamellar structure parameters of the coal samples, and the results of each diffraction peak parameter after the spectra were fitted into peaks as shown in Table 3. Because coal contains various crystalline minerals such as carbonates and sulfates, there are many sharp diffraction peaks with high intensity on the XRD spectra; analysis of the spectra showed that the minerals in the coal were mainly present as SiO_2_, kaolinite Al_2_Si_2_O_5_(OH)_4_, silicate Al_4_(OH)_8_(Si_4_O_10_), and CaCO_3_.

The Scherrer formula and Bragg equation were used [31] to substitute relevant parameters to obtain aromatic structural parameters d_002_, L_a_, L_c_, and others, and the results are shown in Table 4. As can be seen from Table 4, the stacking number of aromatic lamellae of Heiyanquan coal is low, so there are more aliphatic, oxygenated functional groups and side chains, there are relatively more substituted hydrogen atoms, and relatively more fatty side chains attached to the benzene ring.

(1) $$d_{002}=\lambda /2sin\theta_{002}$$

(2) $$L_{a}=K_{2}\lambda /\beta_{100}cos\theta_{100}$$

(3) $$L_{c}=K_{1}\lambda /\beta_{002}cos\theta_{002}$$

(4) $$N_{ave}=L_{c}/d_{002}$$

In Equations (1)–(4), λ is Wavelength of X-rays (nm); θ_002_, θ_100_ is the Bragg angle (°) corresponding to 002 peak and 100 peak; β_002_, β_10_ is the half peak width values (rad) of 002 peak and 100 peak; K_1_, K_2_ is the microcrystalline shape factor, K_1_ = 0.89, K_2_ = 1.84.

### 2.4. ^13^C-NMR Test Result

**Figure 3:**
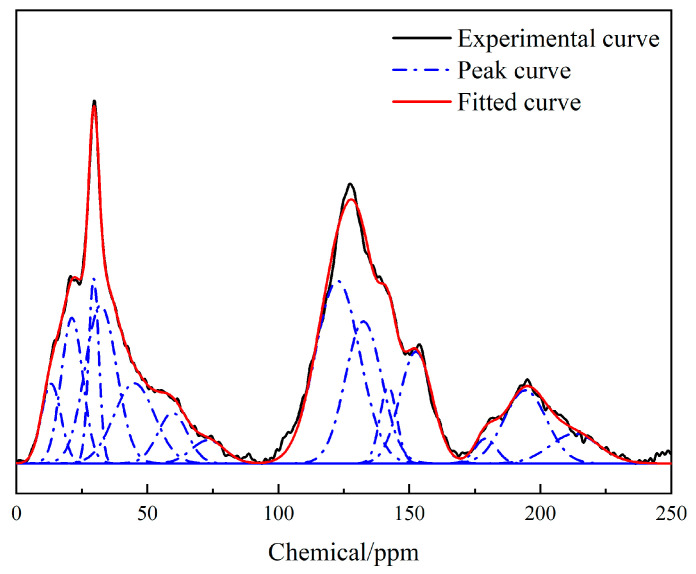
^13^C-NMR spectrum of raw coal.

The nuclear magnetic resonance spectrum of the Heiyanquan coal was roughly divided into the following four sections: (1) fat carbon peak at 0–90 × 10^−6^; (2) aromatic carbon peak at 90–170 × 10^−6^; (3) carbonyl and carboxyl carbon peaks at 170–220 × 10^−6^ [32]. Due to the coal structure’s complexity and ^13^C-NMR’s limitations, it was necessary to conduct peak separation processing of the spectrogram to obtain more structural information. The ^13^C-NMR spectrogram of the sample was peak-fitted using Origin 2019 software, as shown in Figure 3. The specific attribution of each peak position in the ^13^C-NMR spectrum of the coal samples is detailed in Table 5.

According to the ^13^C-NMR peak-fitting results of the Heiyanquan coal sample, its various structural parameters were calculated, as shown in Table 6.

Based on the percentages of each structural parameter of the coal samples in Table 6, the ratio of bridge carbon to perimeter carbon (X_BP_) of the aromatic compounds in the molecular structure of the Heiyanquan coal was calculated thus: X_BP_ = $f_{\alpha }^{B}$/($f_{\alpha }^{H}$+$f_{\alpha }^{P}$+$f_{\alpha }^{S}$) = 0.397. This parameter reflected the average value of the degree of aromatic ring condensation in the coal structure, from which the size of the aromatic cluster was calculated [33].

As shown in Table 6, the aromatic carbon content of the Heiyanquan coal was 57.74%, while the aliphatic carbon content was 42.26%. Given $f_{\alpha }^{S}$ = 3.21, the presence of a small amount of aromatic carbon was substituted by alkyl groups; $f_{\alpha }^{P}$ = 8.97, indicating the presence of a small amount of hydroxyl and ether bonds outside the aromatic carbon; $f_{\alpha }^{B}$ = 12.83, indicating that the bridging aromatic carbon accounted for the largest proportion of the non-protonated aromatic carbon in the aromatic carbon structure of this coal molecule; $f_{\alpha l}^{*}$ = 3.84 and $f_{\alpha l}^{0}$ = 5.60, indicating the aliphatic carbon structure with methyl, methoxy, oxygenated substituent, and oxygenated hypomethyl carbon, and all their content was significantly smaller than that of methylene.

### 2.5. FTIR Test Results

**Figure 4:**
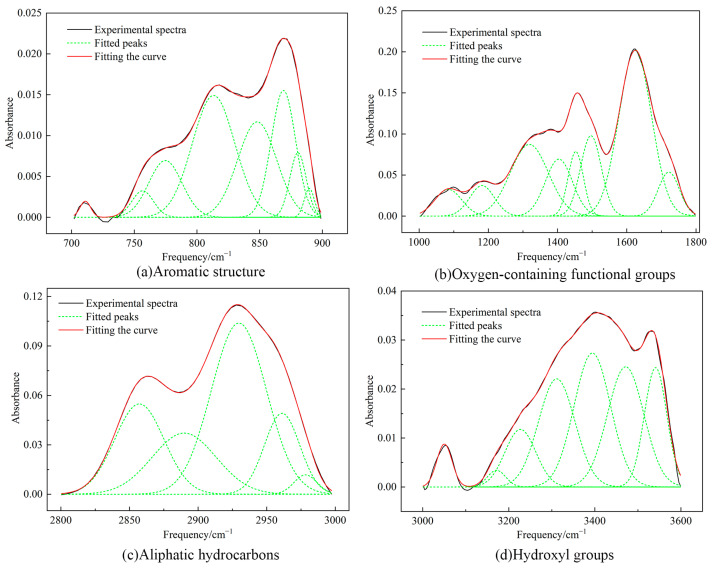
Infrared split-peak fitting of the raw coal.

The absorption peaks of the FTIR spectra of the coal samples were mainly distributed in the following four intervals: (1) the aromatic structure between 700–900 cm^−1^; (2) the absorption vibration of oxygen-containing functional groups between 1000–1800 cm^−1^; (3) the aliphatic hydrocarbons segment between 2800–3000 cm^−1^; and (4) the hydroxyl groups between 3000–3600 cm^−1^ [34]. For a better understanding of the distribution of the functional groups in the coal, infrared split-peak fitting of the raw coal is shown in Figure 4.

The results of the split-peak fitting summarized the functional groups of the coal molecules, as shown in Table 7, Table 8, Table 9 and Table 10. The molecular structure of the Heiyanquan raw coal indicated a significant proportion of hydroxyl groups, mainly from hydroxy ether oxygen, and self-associated –OH. In contrast, CH_2_ and CH accounted for approximately 63%, which was consistent with the results of the ^13^C–NMR analysis. Benzene ring tetrasubstitution dominated the benzene ring substitution with a 75% ratio. The peak area ratio of C=O to C–O in the raw coal was found to be 2:3. In addition, the peak between 3000–3600 cm^−1^ may be related to the presence of clay minerals in the coal, possibly indicating –OH stretching vibrations of hydrogen-bonded hydroxyl groups of water absorbed by clay minerals [35].

### 2.6. Basic Structure of the Coal Molecular Model

#### 2.6.1. Aromatic Structure

The C content of the Heiyanquan coal was 69.85%, with the ratio of bridge carbon to peripheral carbon (X_BP_) of the aromatic compounds at 0.397. This ratio was 0.25 for naphthalene, 0.4 for phenanthrene and anthracene, and 0.38 for 2 × 2 aromatic rings, indicating domination by phenanthrene and anthracene in the aromatic skeleton of the coal. The X_BP_ value of the molecular structure model of the Heiyanquan coal was found to be close to 0.397 by adjusting the type and number of each aromatic structure, as shown in Table 11.

#### 2.6.2. Aliphatic Structure

The aliphatic carbon structure in the coal existed in various forms, including, CH, CH_2_, fatty side chains, and cycloalkanes. A detailed breakdown is presented in Table 7, indicating a fat percentage of 42.26%, with the estimated number of aliphatic carbon, total aromatic carbon, and total carbon atoms in the coal molecule at 74, 101, and 175, respectively. Based on the atomic ratio of the coal sample, there were 125 hydrogen atoms in the Heiyanquan coal molecule.

#### 2.6.3. Heteroatomic Structure

The total number of carbon atoms in the structure of the Heiyanquan coal was tentatively determined as 175. The number of O and N atoms in the coal was estimated at 21 and three, respectively, by combining the elemental analysis with the ratio of each element to C atoms.

The coal samples analyzed in the experiment contained various oxygen-containing functional groups, including carboxyl groups, hydroxyl groups, and ether-oxygen bonds. The corresponding proportion of ether-oxygen bond C–O–C to carbonyl C=O ratio was determined to be 3:2 based on the infrared split-peak fitting results, indicating a dominance of ether-oxygen bonds. Furthermore, the macromolecular model of the Heiyanquan coal contained seven carbonyl groups, ten ether-oxygen bonds, and four hydroxyl groups.

The predominant forms of nitrogen in the coal structure were pyridine-type nitrogen and pyrrole-type nitrogen Combined with the XPS results, it was detected that three nitrogen atoms existed in the Heiyanquan coal, consisting of two pyridyl-type nitrogen and one pyrrole-type nitrogen.

Additionally, organic sulfur in the coal was identified as thiol-thiophenol and thiophene-type sulfur. However, the sulfur mass fraction of the coal was too low (only 0.32%), and it is not discussed further during the analysis.

#### 2.6.4. Coal Molecular Structure Model

**Figure 5:**
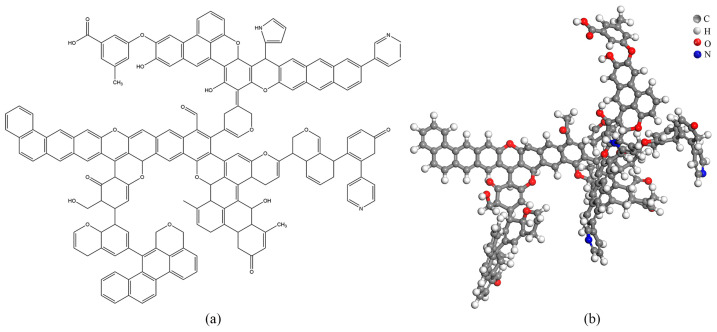
Heiyanquan coal model plane structure (**a**) and three-dimensional construction (**b**).

Based on the experimentally obtained molecular structure parameters of the coal, a planar model of the Heiyanquan coal structure was constructed, and the model was drawn using ChemDraw, based on which the geometric optimization of the molecular model structure and annealing kinetic simulation were performed via Materials Studio (MS) [36]. The planar structure model and the 3D structure are shown in Figure 5, and the structural parameters before the model optimization and after the optimization via annealing simulation are listed in Table 12. 

#### 2.6.5. Coal Molecular Structure Optimization

**Figure 6:**
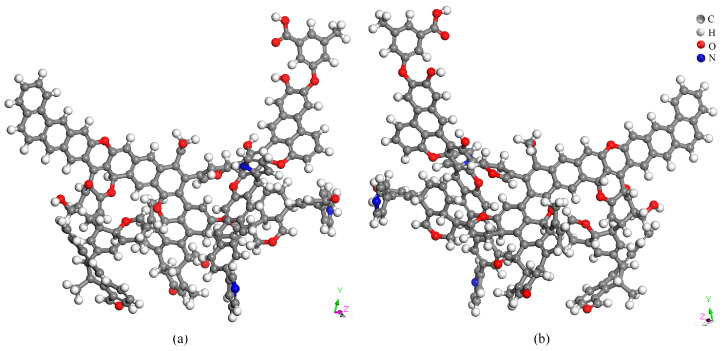
Molecular structure after optimization from different angles (**a**) and (**b**).

The Forcite module in Material Studio 2019 software was utilized for geometric optimization of the constructed Heiyanquan coal structure model, followed by annealing dynamics simulation to obtain the final stable energy state model. Materials Studio was used to optimize and anneal the structural model of the coal samples. The optimized structure model of the coal sample is shown in Figure 6. Table 13 illustrates the energy composition before and after the optimization of the structural model of the Heiyanquan coal. The total energy was 200,974.99 kJ/mol before the optimization and 574.23 kJ/mol after optimization, indicating a substantial decrease in the total energy. In other words, the initial Heiyanquan coal exhibited a relatively unstable structure with molecular-scale defects, emphasizing the necessity for optimization to achieve a molecular structure closely resembling the actual structure of the coal sample [37]. In the optimized model, all energies were reduced to different degrees, with the van der Waals energy experiencing the most significant reduction. Due to the large number of fat side chains in the model of coal molecular structure, substantial torsion occurs in the process of structural optimization, so the torsion energy E_T_ is the largest among the optimized bonding energies, and the Π–Π interaction makes the van der Waals energy E_Van_ dominant among the non-bonding energies. It can be observed that the van der Waals (E_Van_) and torsion energies (E_T_) play an important role in the stability of the optimized structure and the short-range ordering of the aromatic lamellae.

### 2.7. Coal Oxidation Characterization Analysis

#### 2.7.1. TG Test Results and Analysis

(1) Characteristic Temperature and Stage Division

**Figure 7:**
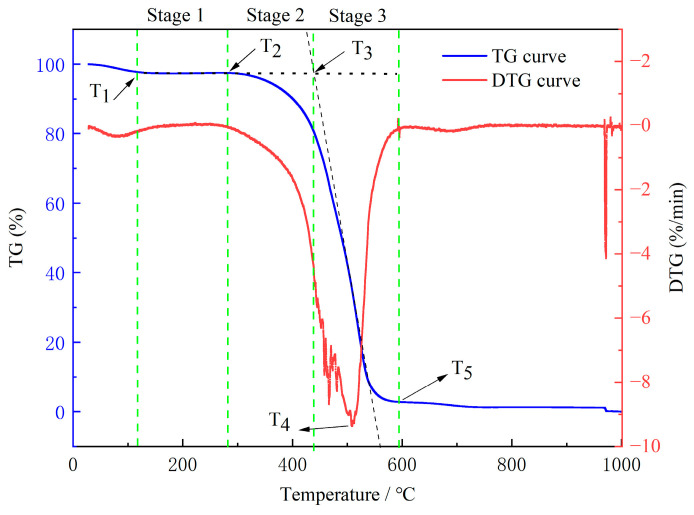
TG/DTG curves of the raw coal.

The TG (weight loss) and DTG (decomposition rate) curves of the experiments are illustrated in Figure 7. Temperature is one of the most important characteristic parameters in spontaneous coal combustion. According to the characteristic mass change in the coal during spontaneous combustion and warming, the following five characteristic temperatures were determined: the inflection point temperature (T_1_), the onset of combustion temperature (T_2_), the ignition temperature (T_3_), the temperature at the maximum burning rate (T_4_), and the burnout temperature (T_5_) [38]. It was observed that these characteristic temperatures increased gradually with rising temperature, which indicated that spontaneous coal combustion was an accelerated heating process.

According to the curve in Figure 7, the oxidation and spontaneous combustion of the coal were divided into the following three stages: stage 1, water loss and drying (T_1_–T_2_); stage 2, oxidation and weight gain (T_2_–T_3_); and stage 3, combustion (T_3_–T_5_).

**Figure 8:**
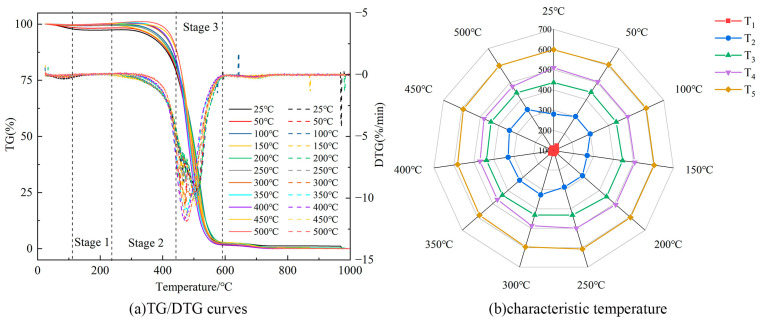
TG/DTG curves and characteristic temperature changes of the Heiyanquan coal.

Figure 8 illustrates the TG/DTG curves and characteristic temperature variations of Heiyanquan coal at different temperatures. As the temperature increases, the reaction process of water loss and the drying stage in the spontaneous combustion of coal becomes longer, while the oxidation and weight gain stage becomes obviously shorter. The change in the combustion stage remains relatively stable due to the increased likelihood of reaction between aromatic hydrocarbons and the aliphatic chain in the molecular structure of the coal as the temperature rises. This accelerates the process of coal combustion, and a large number of reactive groups in the coal are involved in the reaction during the combustion stage, leading to the gradual decrease in T_4_ with the rise in temperature. T_3_ and T_5_ remain basically unchanged, as the active aliphatic hydrocarbon functional group in coal gradually decreases with the increase in temperature, while the demand to maintain the reaction proceeds increases, and the consumption of aromatic hydrocarbons increases, resulting in a more difficult reaction.

(2) Kinetic Parameters

The Coats–Redfern integral method was used to calculate the activation energy. The reaction rate of coal combustion can be obtained from Arrhenius’ law by considering the oxygen combustion reaction of coal as a primary reaction:

(5) $$k=Aexp\left(-E/RT\right)$$

where k is the coal-oxygen rate reaction constant; A is the frequency factor; E is the activation energy, kJ·mol^−1^; T is the reaction temperature K; and R is the gas constant, R = 8.314 J·(mol·K)^−1^.

For general reaction temperature regions and activation energy E values, E/RT ≥ 1 and 1-2RT/E tends to 1. Therefore, Equation (6) is used to calculate the kinetic parameters:

(6) $$\ln\left[-ln\left(1-\alpha \right)/T^{2}\right]=-\left(E/RT\right)$$

**Figure 9:**
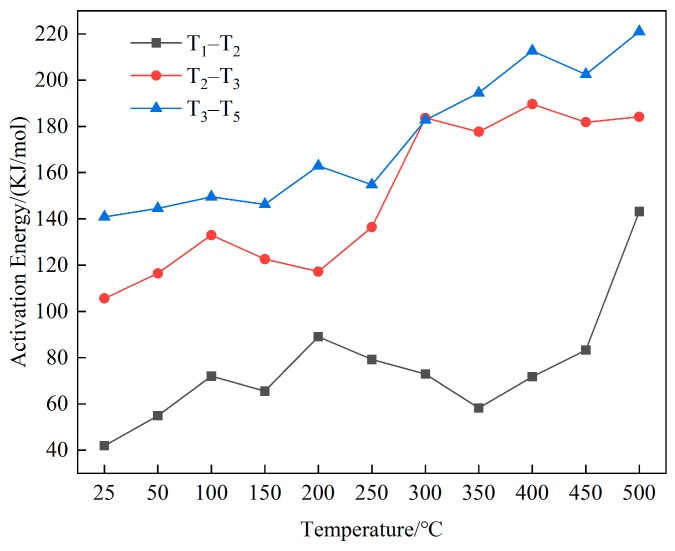
Trend of activation energy in the three stages.

By plotting ln[−ln(1 − α)/T^2^] as the vertical axis and 1/T on the horizontal axis, the slope of the line after linear fitting is the activation energy of the reaction. The trend of activation energy with the temperature rise in the three stages is shown in Figure 9.

In the water loss and drying stage, the activation energy required for the coal was small, but with the increased temperature, the activation energy gradually rose. In the oxidation and weight gain stage, the activation energy of the coal did not change significantly at medium-to-low temperatures but increased significantly at medium-to-high temperatures. This result was because the intermediate products accumulated in the first stage of spontaneous coal combustion began to decompose gradually in the stage of weight gain, thus, generating –OH, –COOH, and other reactive groups, coupled with the high content of oxygen-containing groups, aliphatic hydrocarbons, and hydroxyl groups in the coal each group participated in the reaction, increasing the activity of reactive molecules, and lowering the energy required for the reaction to occur [39]. After the temperature rose to a certain degree, the various types of active functional groups that had previously accumulated were consumed, and the activation energy required to continue the reaction rose significantly.

In the combustion stage, the activation energy value rose with the elevated temperature. The high content of the aromatic structures in the coal led to stable aromatic hydrocarbons in the structure as the core of the molecular structural units of the coal. Since the aromatic hydrocarbons participated in the reaction only at high temperatures, more energy was needed to destroy the aromatic structures, even if the probability of molecular collision was high. Numerous aromatic structures were involved in the reaction in the combustion stage, and the activation energy value was higher in this stage than in the stage of weight gain. It required more energy, and numerous functional groups were involved in the reaction to give off more heat.

#### 2.7.2. FTIR Test Results and Analysis

**Figure 10:**
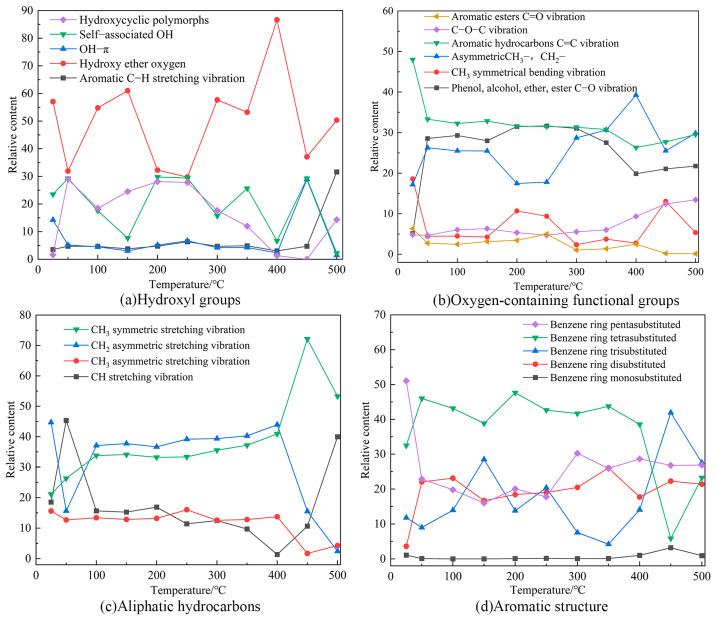
Variation of the functional group content of the coal at different temperatures.

The absorption peak areas of the different functional groups were derived using the integration method via Origin software. The absorption peak areas represent the quantity of a functional group in coal molecules. The changes in the functional groups involved in the oxidation of the coal were divided into the following four main categories: oxygen-containing functional groups; aliphatic hydrocarbons; aromatic structure; and hydroxyl groups [40]. The changes in the functional groups in the coal as a function of the temperature were obtained via comparison. The trends of the peak areas of the functional groups as a function of the temperature are shown in Figure 10.

According to the characteristic temperatures of TG, the temperature-dependent reactions during the coal combustion were divided into the following two stages: (1) 25–300 °C, during which gases such as water, CO_2_, N_2_, and CH_4_ were mainly produced; and (2) 300–500 °C, where the depolymerization and thermal decomposition reactions of the coal mainly occurred, producing gaseous hydrocarbons, CO, and CO_2_. The carbonate minerals release additional CO_2_ during pyrolysis.

Figure 10a shows the changes in hydroxyl groups, with the aromatic ring C–H vibration and OH–Π groups being the lowest, both of which are virtually unchanged until 400 °C, suggesting that both are hardly involved in the coal-oxygen reaction in this stage. Between 25–300 °C, the hydroxyl ether oxygen and the self-associated OH show opposing fluctuating change. Due to water evaporation and oxygen adsorption in the coal, the hydroxycyclic polymorph groups show fluctuating changes, but the overall content increases. Between 300–500 °C, the content of hydroxycyclic polymorphs groups and hydroxy ether oxygen groups increases, while the content of the remaining functional groups fluctuates and the total content decreases.

The variation of oxygen-containing functional groups is illustrated in Figure 10b. The relatively small amount of C=O, C–O–C, and CH_3_ symmetric bending vibration with a small range of variations suggests that these functional groups are weakly active during the coal-oxygen reaction. But at the initial stage of the reaction, the C=O content of the aromatic ester decreases, which is because as the reaction proceeds, the C=O involved in the reaction breaks the chemical bond to form functional groups such as C–O. As the combustion reaction intensifies, the C–O–C content increases and the overall CH_3_ content decreases, because the aliphatic side chain breaks during combustion, generating a large amount of volatiles to be released.

Figure 10c shows that between 25–300 °C the content of CH stretching vibrations and CH_2_ asymmetric stretching vibrations decrease slightly, and the content of CH_2_ asymmetric stretching vibrations and CH_3_ symmetric stretching vibrations increase with increasing temperature. The content of aliphatic groups in coal samples is relatively large, but their activity is relatively low during coal oxidation. During 300–500 °C, the content of CH stretching vibration and CH_3_ symmetric stretching vibration increases to 90%, and the content of CH_2_ and CH_3_ asymmetric stretching vibration decreases significantly, which is due to the reaction of aliphatic hydrocarbons with oxygen to generate a large number of gases, which reduces their content. 

Figure 10d demonstrates that the content of the aromatic structure functional groups varies less throughout the reaction stage, which is because the benzene ring does not decompose in the low-temperature stage, and only participates in the reaction under high-temperature conditions. Between 300–500 °C, the content of benzene ring tetrasubstituents decreases significantly with increasing reaction temperature, the content of benzene ring pentasubstituents and benzene ring disubstituents does not change much, and the content of benzene ring trisubstituents shows fluctuating changes.

#### 2.7.3. HRTEM Test Results and Analysis

**Figure 11:**
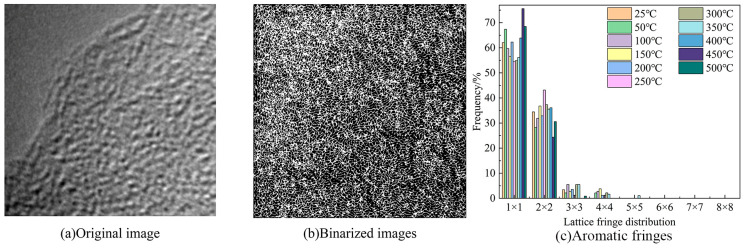
HRTEM image analysis of the Heiyanquan coal.

The HRTEM microscopic images were processed using the Fourier-inverse Fourier transform, image homogenization, and binarization via Digital Micrograph 3.11.2 software. According to the classification method, the lattice stripe length corresponded to the size of the aromatic carbon layer, and its size distribution in the coal was statistically calculated and processed via MATLAB R2019a software, to obtain the length distribution of the lattice stripes of the coal, as shown in Figure 11.

As shown in Figure 11, the stripe length distribution of Heiyanquan coal is dominated by 1 × 1 and 2 × 2, and these two categories account for more than 90% of the total number of stripes, and this result corresponds to the molecular structure of the coal constructed. In Heiyanquan coal, most of the streaks exist in the form of 1 × 1, such as naphthalene, anthracene, and phenanthrene. According to the TG experiment, this combustion process was divided into two parts. In the first stage of combustion at 25–300 °C, the content of aromatic lamellae of 1 × 1 decreases with increasing temperature, while the content of aromatic lamellae of 2 × 2 is opposite to that; at 300–500 °C, the content of aromatic lamellae of 1 × 1 increases with increasing temperature, and the content of aromatic lamellae of 2 × 2 gradually decreases. This is because, at the low-temperature stage, the more stable structure of the aromatic nucleus structure will not participate in the reaction, while with the increase in temperature the degree of deterioration of the coal is intensified, which leads to the change of the aromatic lamellae within the coal due to the decomposition of the oxygen-containing functional groups adjacent to the aromatic structure, which form gaseous products, such as CO_2_. In addition, the breakage of ether bonds attached to the aromatic rings results in the formation of some of the aromatic lamellae with a lower ring number [41].

## 3. Materials and Methods

### 3.1. Materials

The coal samples were collected from the Heiyanquan mining area in Xinjiang, crushed, ground, and sieved. The resultant samples, with a diameter of 80–100 mesh (0.178–0.150 mm) were used as the experimental coal samples.

### 3.2. Test Methods

#### 3.2.1. Proximate and Ultimate Analyses

A Vario EL cube elemental analyzer (Vario EL cube, Elementar, Langenselbold, Germany) was used, and the proximate and ultimate analyses of the coal samples were performed according to the Chinese National Standards GB/T212–2008 [42] and GB/T476–2008 [43].

#### 3.2.2. X-ray Photoelectron Spectroscopy (XPS)

X-ray photoelectron spectroscopy was analyzed via an XPS (Thermo Fisher Scientific ESCALAB250Xi, Thermo Fisher, Waltham, MA, USA). To determine the occurrence form of C, O, N, and S atoms in the coal sample, the experimental conditions were set as follows: monochromatic AL Kα emission source (hv = 1486.6 eV), power of 200 W, and energy analysis range of 0–5000 eV, with C 1s (284.6 eV) as the standard for charge correction.

#### 3.2.3. X-ray Diffraction (XRD)

A D8 Advance X-ray powder diffractometer produced by Bruker (AXS GmbH, Karlsruhe, Germany) was used for the measurement. The Voltage was 60 kV. The current was 50 mA, and the scanning speed was 1500/min.

#### 3.2.4. Nuclear Magnetic Resonance Carbon Spectrum (^13^C-NMR)

Nuclear magnetic resonance carbon spectrum was conducted via an AVANCE III 600 MHz, Bruker, Switzerland at a resonance frequency of 150.91 MHz. Spectra were recorded with a 3.2 mm probe at 25 °C at a rotation rate of 15 kHz. The experimental delay time, contact time, and number of scans were set to 3 s, 2 ms, and 2000, respectively.

#### 3.2.5. Fourier Transform Infrared Spectrometer (FTIR)

A Fourier transform infrared spectrometer (VERTEX 70, BRUKER, Karlsruhe, Germany) was used at a resolution of 4.0 cm^−1^ by scanning the sample 64 times in a wavenumber range of 400–4000 cm^−1^.

#### 3.2.6. Thermogravimetry (TG)

The thermogravimetry differential thermal synchronous analyzer STA7300 produced by Hitachi electronics (Tokyo, Japan) was used as the experimental instrument to monitor the mass loss of the coal during its combustion at 30–1000 °C with a heating rate of 10 °C/min.

#### 3.2.7. High Resolution Transmission Electron Microscopy (HRTEM)

High Resolution Transmission Electron Microscopy (HRTEM) was carried out via a Jem-2100 HRTEM, Tokyo, Japan Electronics, Japan under the measurement conditions of an accelerating voltage of 200.0 kV, a point resolution of 0.19 nm, and a lattice resolution of 5 nm.

## 4. Conclusions

1. The Heiyanquan coal has the molecular empirical formula C_175_H_125_O_21_N_3_ and is characterized by a high aromatic carbon content (57.74%), with the aliphatic carbon content exceeding the CH and CH_2_ contents. Themolecular structure of the coal includes two pyridines and one pyrrole as heteroatoms. Upon geometric optimization and annealing simulation, there was a substantial decrease in total energy, with the Van der Waals energy playing aprominent role.
2. As the temperature increased, the starting combustion temperature (T_2_) gradually rosewhile the temperature at the maximum combustion rate (T_4_) decreased. The remaining characteristic temperatures remained nearly constant. During the stage of water loss and drying, the activation energy required for the coal was small but increased with rising temperature. In the oxidation and weight gain stage, the activation energy did not significantly change at low-to-medium temperatures but rose significantly at high-to-medium temperatures. In the combustion stage, the activation energy increased with the temperature.
3. With the rise in reaction temperature, the C=C content decreased significantly, and the C–O content exhibited an increasing trend and higher activity. The content of benzene ring substituents varied considerably and mainly occurred in the high-temperature stage. The hydroxy ether oxygen groups demonstrated a broader range of variation due to higher activity.

The aromatic rings of the aromatic carbon layer in the molecular structure of the Heiyanquan coalprimarily consist of 1 × 1 and 2 × 2, together accounting for more than 90% of the total, which remained stable with the increasing combustion temperature. Between 25–300 °C, the content of aromatic lamellae of 1 × 1 decreases with increasing temperature; between 300–500 °C, it increases with increasing temperature, and 2 × 2 demonstrated the opposite trend.

## Figures and Tables

**Table 1:** Proximate and ultimate analyses of the Heiyanquan coal.

Proximate Analysis/(Mass)%				Ultimate Analysis/(Mass)%					H/C	O/C	N/C	S/C
M_ad_	A_ad_	V_daf_	FC_ad_	C_ad_	H_ad_	O_ad_	N_ad_	S_td_				
2.68	9.65	38.15	49.52	69.85	4.90	10.90	1.70	0.32	0.84	0.12	0.02	0.0017

Note: M: moisture; A: ash; V: volatiles matter; FC: fixed carbon; C: carbon; S: full sulfur; H: hydrogen; O: oxygen; N: nitrogen.

**Table 2:** Peak-fitted XPS test results of the Heiyanquan coal.

Elemental Peak	Binding Energy/eV	Attribution	Relative Content/%
C (1s)	284.72	C–C, C–H	61.64
	285.36	C–O, C–O–C	33.74
	286.81	C=O	2.27
	289.83	COOH	2.34
O (1s)	532.52	C=O	47.35
	533.17	C–O	52.44
	538.98	adsorbed oxygen	0.21
N (1s)	399.01	N–6	44.50
	399.93	N–6	13.17
	402.23	N–Q	11.55
	400.49	N–5	15.03
	400.97	N–5	11.55
S (2p)	163.58	Mercaptan thiophenol	6.01
	164.77	Thiophene type sulfur	56.53
	167.78	Sulfoxide sulfur	26.74
	169.58	Inorganic sulfur	10.72

**Table 3:** Curve-fitted XRD of Heiyanquan coal.

Peak	2θ/°	FWHM	Peak Type
γ	13.12	7.64	Gaussian
002	22.81	8.81	Gaussian
100	43.35	10.76	Gaussian

**Table 4:** Microcrystalline structural parameters of Heiyanquan coal.

Sample	d_002_	L_c_	L_a_	N_ave_
Heiyanquan Coal	3.90	9.10	16.25	2.33

**Table 5:** Structure parameters of ^13^C-NMR peak fitting.

Peak	Centre	Peak Types	Relative Area/%	Attribution
1	13.29	Gaussian	3.84	R–CH_3_
2	21.21	Gaussian	7.77	RCH_3_–CH_2_
3	29.55	Gaussian	4.68	CH_2_
4	32.22	Gaussian	12.35	CH_2_
5	45.06	Gaussian	8.02	C,CH
6	59.63	Gaussian	3.69	O–CH_3_,O–CH_2_
7	73.16	Gaussian	1.92	O–CH
8	122.59	Gaussian	20.15	Ar–H
9	132.53	Gaussian	12.83	C–C
10	142.00	Gaussian	3.21	Ar–C
11	152.56	Gaussian	8.97	Ar–O
12	179.16	Gaussian	1.32	COOH
13	194.27	Gaussian	7.67	C=O
14	213.10	Gaussian	3.58	C=O

**Table 6:** Percentage of the structural parameters of the Heiyanquan coal.

Parameters	$f_{a}$	$f_{\alpha }^{C}$	$f_{\alpha }^{\prime }$	$f_{\alpha }^{H}$	$f_{\alpha }^{N}$	$f_{\alpha }^{P}$	$f_{\alpha }^{S}$	$f_{\alpha }^{B}$	$f_{\alpha l}$	$f_{\alpha l}^{*}$	$f_{\alpha l}^{H}$	$f_{\alpha l}^{0}$
Percentage/%	57.74	12.58	45.16	20.15	25.01	8.97	3.21	12.83	42.26	3.84	32.82	5.60

Notes: $f_{a}$—total aromatic carbon; $f_{\alpha }^{C}$—carbonyl or carboxyl group; $f_{\alpha }^{\prime }$—aromatic C; $f_{\alpha }^{N}$—nonprotonated and aromatic; $f_{\alpha }^{H}$—protonated and aromatic; $f_{\alpha }^{P}$—aromatic C bonded hydroxyl or ether oxygen; $f_{\alpha }^{S}$—alkylated aromatic; $f_{\alpha }^{B}$—aromatic bridgehead C; $f_{\alpha l}$—total sp3 hybridized; $f_{\alpha l}^{*}$—CH_3_ or nonprotonated; $f_{\alpha l}^{H}$—CH or CH_2_; $f_{\alpha l}^{0}$—aliphatic C bonded oxygen.

**Table 7:** Hydroxyl groups functional group parameters of Heiyanquan coal.

Peak	Centre	Relative Area/%	Area	Attribution
1	3050.55	3.54	0.39	Aromatics C–H stretching vibration
2	3171.68	1.63	0.18	Hydroxycyclic polymorphs
3	3227.06	9.55	1.06	Hydroxy ether oxygen
4	3311.53	21.24	2.35	Stretching vibration of secondary amine –NH
5	3394.25	26.29	2.91	Hydroxy ether oxygen
6	3471.64	23.51	2.60	Self-associated OH
7	3541.16	14.24	1.58	OH–Π

**Table 8:** Aliphatic hydrocarbons functional group parameters of Heiyanquan coal.

Peak	Centre	Relative Area/%	Area	Attribution
1	2857.17	21.11	2.47	CH_3_ symmetric stretching vibration
2	2889.83	18.53	2.17	CH stretching vibration
3	2929.75	44.73	5.24	CH_2_ asymmetric stretching vibration
4	2961.20	13.53	1.59	CH_3_ asymmetric stretching vibration
5	2978.31	2.1	0.25	CH_3_ asymmetric stretching vibration

**Table 9:** Oxygen-containing functional groups parameters of Heiyanquan coal.

Peak	Centre	Relative Area/%	Area	Attribution
1	1084.34	4.80	3.12	C–O–C vibration
2	1181.80	5.14	3.34	Phenol, alcohol, ether, ester C–O vibration
3	1318.27	18.57	12.07	CH_3_ symmetric bending vibration
4	1403.86	10.51	6.83	Stretching vibration of alkyl–CH_2_, –CH_3_, C–H
5	1452.62	6.68	4.34	Stretching vibration of alkyl–CH_2_, –CH_3_, C–H
6	1496.52	11.34	7.37	Aromatic hydrocarbons C=C vibrations
7	1624.34	36.64	23.82	Aromatic hydrocarbons C=C vibrations
8	1721.38	6.32	4.11	Aromatic esters C=O vibration

**Table 10:** Aromatic structure functional groups parameters of Heiyanquan coal.

Peak	Centre	Relative Area/%	Area	Attribution
1	710.96	1.07	0.02	Benzene ring monosubstituted
2	756.67	3.60	0.07	Benzene ring disubstituted
3	774.81	11.80	0.23	Benzene ring trisubstituted
4	813.27	32.48	0.63	Benzene ring tetrasubstituted
5	848.02	23.20	0.45	Benzene ring tetrasubstituted
6	868.96	19.44	0.38	Benzene ring tetrasubstituted
7	880.58	6.61	0.13	Benzene ring pentasubstituted
8	889.59	1.80	0.03	Benzene ring pentasubstituted

**Table 11:** Aromatic structure in the Heiyanquan coal model.

Aromatic Structure	Number	Aromatic Structure	Number
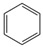	2	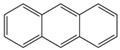	1
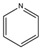	2	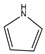	1
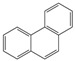	1	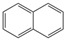	1
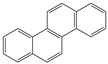	1	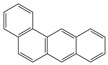	1

**Table 12:** Structural parameters of the Heiyanquan coal model.

Molecular Empirical Formula	Molecular Weight	Chemical Composition (%)			
		C	H	O	N
C_175_H_125_O_21_N_3_	2603	80.68	4.61	12.91	1.61

**Table 13:** Energy changes of structural optimization of Heiyanquan coal (kJ/mol).

Project	Total Energy	E_B_	E_A_	E_T_	E_H_	E_Van_	E_I_	E_E_
Initial	200,974.99	778.29	110.03	308.13	0.00	200,308.95	18.67	−549.08
Final	574.23	89.55	87.99	123.03	−0.01	435.54	2.43	−164.30

Notes: E_B_ = E_Bond_; E_A_ = E_Angle_; E_T_ = E_Torsion_; E_H_ = E_Hydrogen bond_; E_Van_ = E_van der Waals_; E_I_ = E_inversion_; E_E_ = E_electrostatic._

## Data Availability

Data are contained within the article.
